# Predictors of willingness to pay for physical activity of socially vulnerable groups in community-based programs

**DOI:** 10.1186/s40064-015-1336-5

**Published:** 2015-09-19

**Authors:** Marion C. Herens, Johan A. C. van Ophem, Annemarie M. A. E. Wagemakers, Maria A. Koelen

**Affiliations:** Social Sciences Group, Chair Group Health and Society, Wageningen University and Research, Hollandseweg 1, Postbus 8130, 6700 EW Wageningen, The Netherlands; Social Sciences Group, Chair Group Economics of Consumers and Households, Wageningen University and Research, Hollandseweg 1, Postbus 8130, 6700 EW Wageningen, The Netherlands

**Keywords:** Sport and physical activity, Community-based, Willingness to pay, Socially vulnerable groups, Health-related quality of life

## Abstract

Willingness to pay (WTP) is used to assess individuals’ value attribution to health-related quality of life interventions. Little is known about predictors of WTP for sport and physical activity in socially vulnerable groups in community-based physical activity (CBHEPA) programs. This study addresses the questions: What is the WTP for sport and physical activity of participants in CBHEPA programs, expressed in WTP_money_ and WTP_time_? Which factors predict WTP_money_ and WTP_time_? From the literature, predictors for WTP for sport and physical activity were identified: (1) personal and socio-economic predictors: income, education, age, and ethnic origin, (2) health-related predictors: perceived health, life satisfaction, sense of coherence, self-efficacy, (3) sport and physical activity-related predictors: duration and frequency of participation, leisure-time sport or physical activity, sport club membership, enjoyment, and membership fee. Data were gathered for WTP_money_ and WTP_time_ (n = 268) in 19 groups in an evaluation study of CBHEPA programs. Ordered probit was used for analyses. WTP_money_ was a monthly average of €9.6. WTP_time_ was on average 17.6 min travel time. Income was found as predictor for both WTP_money_ and WTP_time_. Other predictors for WTP_money_ were: duration and frequency of program participation, enjoyment, and (former) sport club membership. Low income and younger age were found as predictors for WTP_time_. Predictors for WTP_money_ are related to income and sport and physical activity experiences, for WTP_time_ to income and age. Short-term program satisfaction is probably more decisive for WTP_money_ than long-term perspectives of improving health-related quality of life.

## Background

Physical inactivity has been identified by the World Health Organisation as the fourth leading risk factor for global mortality, causing globally an estimated 3.2 million deaths per year (GAPA 2012; WHO 2012). Health disorders associated with inactivity, including impaired health-related quality of life as well as direct and indirect economic costs, exert a substantial burden on societies and health systems (Craig et al. 2012). In the Netherland, socially vulnerable groups, e.g. those with low socio-economic status (SES), unemployed or of non-Dutch origin, are less engaged in sport and physical activity than higher SES groups (Wendel-Vos et al. 2009; Hildebrandt et al. 2013). In response to the observed inequalities, Dutch policy has been to promote community-based health-enhancing physical activity (CBHEPA) programs in order to improve the health and wellbeing of socially vulnerable groups (Ministry of Health Welfare and Sports 2006, 2012). Approximately €60 m are spent on campaigns, research, and institutions to promote healthy and active lifestyles, and healthy social and physical environments (Post et al. 2010; De Wit et al. 2010). In 2010 (local) sports-related government expenditures were ca. €3.5 bn, spent on exploitation costs, maintenance of sports facilities and subsidy schemes enhancing sport and physical activity (Centre for Policy-related Statistics 2013). A substantial portion of the subsidy schemes is dedicated to enhancing physical activity behaviour in socially vulnerable groups. Not much is known, however, about the extent to which socially vulnerable groups themselves are able and willing to invest in sport or physical activity in order to achieve active and heathy lifestyles.

Over the past two decades, the contingent valuation method (CVM) asking people’s stated preferences for a good or a health service (Morris et al. 2007), is being used more often in health economics research to assess value attribution at individual level to health-related quality of life interventions (Klose 1999; Donaldson and Shackley 2003; Drummond et al. 2005; Olsen et al. 2004; Cawley 2004; Lorgelly et al. 2010; Murphy et al. 2012). CVM assumes a direct relationship between the amount of money or time invested and the health benefits experienced (Borghi and Jan 2008). Assessment of willingness to pay (WTP) is a relatively easy CVM to study perceived benefits at individual level of CBHEPA programs. WTP reflects the extent to which people are willing to pay for positive health improvements (Drummond et al. 2005; Remonnay et al. 2008). Usually, WTP is expressed in monetary terms (WTP_money_). Willingness to spend time travelling to sport and physical activity (WTP_time_)—which in transportation models is seen as a disutility that should be minimised—should be regarded as an additional estimator of positive value attribution (Dijst and Vidakovic 2000), since it expresses willingness to make an effort to participate.

Relevant literature on WTP for recreational sport and physical activity is, however, fairly limited. Johnson et al. (2007) argued that published CVM studies of sports public goods have mostly focused on WTP for professional or spectator sports. The fact that governments also subsidise other sport and physical activities, such as amateur and recreational sport or CBHEPA programs, is usually not taken into account. The underlying idea of these subsidy schemes is that participation in sport and recreational physical activities is supportive to the development of social capital by contributing to community bonding, hence enhancing quality of life in a community (Putnam 2000; Lindström et al. 2001; Skinner et al. 2008). It may also improve the health and well-being of participants and reduce health-care costs (Hawe and Shiell 2000; Johnson et al. 2007).

In view of these expected societal benefits, it is unclear whether predictors for WTP for health improvements also predict WTP for sport and physical activity in CBHEPA programs. In this study we use WTP as a particular measure to assess value attribution to the experienced benefits of CBHEPA programs by individual participants, in addition to physical activity and health-related outcome measures, in order to contribute to a broader recognition of the (non)sense of government investments in CBHEPA programs. In order to assess the capacity and willingness to invest in sport and physical activity of socially vulnerable groups, our study addresses the following questions: What is the WTP for sport and physical activity of participants in CBHEPA programs in terms of money and time (WTP_money_ and WTP_time_)? Which factors predict WTP_money_ and WTP_time_?

### Predictive factors for WTP for sport and physical activity

Little is known about predictors for WTP for sport and physical activity. WTP values drawn from a CVM survey are determined by personal and behavioural characteristics of the respondent and characteristics of the service specified (Smith 2003). Regarding personal and behavioural characteristics, studies on WTP for health improvements indicate that personal and socio-economic factors as well as health-related quality of life factors are relevant predictors (Bauman et al. 2002; Hagger et al. 2002; Rhodes et al. 2007). Regarding service characteristics, sport and physical activity behaviour and program-related factors may be relevant predictors. For our study, we assume that factors predicting health-related quality of life may be relevant for predicting WTP for health improvements, and factors predicting WTP for health improvements may be relevant for WTP for sport and physical activity.

1. *Personal and socio*-*economic predictors* relate to an individual’s non-behavioural conditions, setting the boundaries for individual demand. Some studies suggest that WTP is positively related to *income* (Donaldson et al. 1997; Romé et al. 2010), others report no significant relationships (Johannesson and Johansson 1997; Olsen and Smith 2001). In line with a utilitarian perspective, WTP for sport and physical activity is expected to increase with increasing income. Some studies also suggest that WTP is positively related to *educational level* (Romé et al. 2010). More highly educated people are generally more *health literate*, i.e. more knowledgeable on healthy lifestyles and potential risk factors (Ross and Wu 1995). Some studies suggest that WTP is negatively related to *age*, indicating that older people are less willing to pay for health improvements than younger people (Johannesson and Johansson 1997; Krupnick et al. 2002; Romé et al. 2010). In addition, socially vulnerable groups tend to become less healthy and active with increasing age (WHO 2006; Cockerham 2007). Studies on the relation between WTP and *ethnic origin* seem scarce. A negative relationship between WTP and ethnic origin can be assumed, since ethnic origin is related to impaired health (Bos et al. 2004; Pampel et al. 2010) and higher levels of physical inactivity (Crespo et al. 2001; Hildebrandt et al. 2013).
2. *Health*-*related quality of life predictors* relate to an individual’s behaviour and perceived health benefits. Although many instruments, consisting of different components, have been developed to measure health-related quality of life (Bowling 2005), less is known about the relation of each component to WTP for health improvements or sport and physical activity. Components of health-related quality of life that may be relevant for WTP for sport and physical activity are perceived health status (Rütten et al. 2001; Van Stralen et al. 2009), life satisfaction (Downward and Rasciute 2011; Lehnert et al. 2012), the ability to cope with life stressors (Antonovsky 1996; Van Stralen et al. 2009), and self-efficacy relating to physical activity behaviour (Marcus et al. 1992; McAuley and Blissmer 2000; Nickel and Spink 2010). Several studies suggest a positive relationship between WTP for health improvements and *perceived health* status (Donaldson and Shackley 2003; Bayoumi 2004; Borghi and Jan 2008; Victoor et al. 2012), whereas others report no significant relationships (Donaldson 1999). A positive relationship between WTP for health improvements and *life satisfaction* can be expected, since life satisfaction is positively related to health-related quality of life and physical activity. Furthermore, we expect a positive relationship between WTP for health improvements and the ability to cope, or *sense of coherence* (SoC). SoC relates to the way people cope with life stressors and is highly correlated with health-related quality of life (Eriksson and Lindström 2007). Similarly, we expect a positive relationship between WTP and *self*-*efficacy*, i.e. one’s confidence in one’s ability to manage and succeed in specific situations (Bandura 1995), since previous studies show that self-efficacy is positively related to health-related quality of life and physical activity (Marcus et al. 1992; McAuley and Blissmer 2000; Hagger et al. 2002; Bauman et al. 2002; Van Stralen et al. 2009). To our knowledge, however, no previous studies include life satisfaction, sense of coherence, or self-efficacy in WTP research.
3. *Sport and physical activity*-*related predictors* relate to individual behaviour in relation to CBHEPA program characteristics. Recreational literature based on experience use theory suggests that WTP is positively related to *duration and frequency of participation* in a certain activity or program (Kyle et al. 2006; López-Mosquera and Sánchez 2013). Some studies suggest that WTP is positively related to experiences in *leisure*-*time sport* and (*former*) *sports club membership* (Pawlowski et al. 2009; Prins et al. 2010; Downward and Rasciute 2011). People who are or were member of a sport club are more willing to pay for leisure-time sport and physical activity than people with no history in sports (Bauman et al. 2002), and are good estimators of the costs. McCarville (1991) indicates that the level of membership fee can be regarded as the reference fee. In our study, we also include *enjoyment* as a variable, since some studies suggest that people engage in sport and physical activity for pleasure rather than for health benefits (Henderson 2009; Mullen et al. 2011). Therefore, we expect a positive relation between enjoyment and WTP. To our knowledge, no previous studies include enjoyment in WTP research.

Based on this overview, the expected relations between the main predictive factors and WTP for sport and physical activity are summarised in Table 1.

**Table 1:** Summary of expectations for WTP for sport and physical activity

Cluster	Predicting factor	Known predictor for health-related quality of life and physical activity	Known predictor WTP health improvements	Expectation
Personal and socio-economic	Income	*+*	*+/−*	1. Income is positively related to WTP_money_ and WTP_time_
	Educational level	+	+	2. Educational level is positively related to WTP_money_ and WTP_time_
	Age	*+*	*+/−*	3. Age is negatively related to WTP_money_ and WTP_time_
	Ethnic origin	+	?	4. Non-Dutch origin is negatively related to WTP_money_ and WTP_time_
Health-related quality of life	Perceived health status	+	+	5. Individual perceived health status is positively related to WTP_money_ and WTP_time_
	Life satisfaction	+	?	6. Life satisfaction is positively related to WTP_money_ and WTP_time_
	Sense of coherence	+	?	7. Sense of coherence is positively related to WTP_money_ and WTP_time_
	Self-efficacy	+	?	8. Self-efficacy is positively related to WTP_money_ and WTP_time_
Sport and physical activity	Duration	+	?	9. Duration of participation in the CBHEPA program is positively related to WTP_money_ and WTP_time_
	Frequency	+	?	10. Frequency of participation is positively related to WTP_money_ and WTP_time_
	Physical activity enjoyment	+	?	11. Physical activity enjoyment is positively related to WTP_money_ and WTP_time_
	Leisure-time physical activity	+	?	12. Additional leisure-time physical activity is positively related to WTP_money_ and WTP_time_
	Leisure time sport	+	?	13. Additional leisure-time sport is positively related to WTP_money_ and WTP_time_
	Sports club membership	+	+	14. (Former) sports club membership is positively related to WTP_money_ and WTP_time_
	Membership fee	?	?	15. Paying membership fee is positively related to WTP_money_

+, known relation; −, known lack of relation; ?, unknown relation

## Methods

### Participants

We studied respondents’ WTP_money_ and WTP_time_ in on-going Dutch CBHEPA programs, summarised under the denominator ‘communities on the move’ (CoM). CoM was developed and disseminated by the Netherlands Institute for Sports and Physical Activity (NISB) from 2003 to 2012. Since 2012, there has been an on-going evaluation study of CoM (Herens et al. 2013). CBHEPA groups were recruited to participate in the evaluation study in collaboration with NISB and local CBHEPA program representatives (purposive sampling). CBHEPA groups were selected on the basis of their participants’ socio-economic criteria (income, education, employment status). A total of 268 respondents were included, active in 19 CBHEPA groups (10–20 participants) distributed over seven Dutch municipalities. Assuming an average group size of 15, the estimated response rate was 94 %.

### Data collection

Standardised paper-and-pencil questionnaires were developed for evaluating CoM. Data collection for WTP_money_ and WTP_time_ formed an integral part of the standardised questionnaire. WTP_money_ and WTP_time_ were measured using ordinal closed-ended questions. WTP_money_ was measured as the maximum amount (in whole euro’s) people were willing to spend monthly on sport and physical activity [nine-point scale: (1) 0 euro; (2) 1–5 euro; (3) 6–10 euro; … (9) more than 35 euro, namely ….]. WTP_time_ was measured as the maximum time (in minutes) people were willing to spend on travel time to the sport venue (Pawlowski et al. 2009) [nine-point scale: (1) 0 min; (2) 1–5 min; (3) 6–10 min; … (9) more than 35 min, namely …]. The closed-ended data collection was chosen, based on the assumption that it provided for simplicity and uniformity, suitable for use in diverse socially vulnerable groups in CBHEPA programs.

Data on socio-economic indicators (age, income, education, employment status, living conditions) were measured in accordance with standardised questions of the Local and National Monitor Public Health in the Netherlands (National Institute for Public Health and the Environment (RIVM) 2005).

Health-related quality of life data were measured using: a visual analogue scale for perceived health (EQ-VAS), ranging from 0 to 100 (The EuroQol Group 1990); Cantril’s ladder for life satisfaction, ranging from 0 to 10 (Cantril 1965; Peters et al. 2012); and the SoC three-item, three-point scale for sense of coherence (Eriksson and Lindström 2005; Olsson et al. 2009). Questions were: ‘Do you usually see solutions to problems and difficulties that other people find hopeless?’ (manageability), ‘Do you usually feel that your daily life is a source of personal satisfaction?’ (meaningfulness) and ‘Do you usually feel that the things that happen to you in your daily life are hard to understand?’ (comprehensibility).

Sport and physical activity behaviour were measured using the validated Short Questionnaire for Sport and Physical Activity (SQUASH), measuring self-reported work-related, domestic, leisure-time and sport-related physical activities in minutes per week (Wendel-Vos et al. 2003; De Hollander et al. 2012). Physical activity enjoyment was measured using a nine-item, five-point scale, translated and adapted from the Physical Activity Enjoyment Scale (Mullen et al. 2011). Statements were for example: ‘When I do exercise or sports, I enjoy it’, or ‘When I do exercise or sports, I feel bored’. Self-efficacy for physical activity behaviour was measured using a six-item, five-point scale (Bandura 2006). Statements were for example: ‘I am confident that I am able to continue to participate in the physical activity program during the coming months’, and ‘I am confident that I am able to continue to participate in the physical activity program when I am tired’.

Questionnaires were individually filled in during or after a group training session at the sports venue. Informed consent was arranged orally on the spot and confirmed in writing. The researcher explained the purpose of the study at each session. Both the researcher and trained assistants helped respondents who had difficulty filling out the questionnaire by giving instructions or by adopting an interview style. The number of assistants varied with group composition: from one for groups with only Dutch native speakers to a maximum of five in groups with migrant respondents. Dutch was the working language, since ethnic diversity within groups was large (>10 countries of origin). Interpretation, if needed, was provided by an assistant or a fellow group member from a similar background, sufficiently proficient in Dutch. Completion of the questionnaire took on average 30–35 min. After filling out the questionnaire, respondents received a small treat.

### Data analysis

The dependent variables WTP_money_ and WTP_time_ were recoded into seven categories. Assumptions for normality were explored. The income variable was recoded and tested with a Pearson Chi square test to check for the assumption that it could be used as independent test variable, despite the fact that 28.1 % of the respondents did not specify income (not knowing, not wanting to). There was no significant association between WTP_money_ categories and whether or not respondents had specified their income (χ^2^ = 6.208; p > 0.05); this led to the conclusion that income could be used in the model.

The variables for age and education were recoded into categories, and assumptions for normality were checked. The scale variables Physical Activity Enjoyment Scale (Cronbach’s α = 0.87) and self-efficacy (Cronbach’s α = 0.69) were calculated, recoding each item into the same direction, and excluding system missing values. An ordered probit analysis was used (SPSS22) to assess factors predicting WTP_money_ and WTP_time_. The different expectations for WTP_money_ and WTP_time_ were tested, using p < 0.10 as the upper limit for statistical significance (Greene 2003; Jackson 2008).

The authors declare that the study was conducted in accordance with general ethical guidelines for behavioural and social research in the Netherlands. Participation was on a voluntary basis and guarantees of anonymity were given prior to each data collection session.

## Results

### Descriptive statistics

A total of 268 respondents were included, 86.6 % women and 13.4 % men, with a mean age of 58.6 years old (*sd* 14.0). One-third of the respondents (35.4 %) were of Dutch origin, 64.6 % of non-Dutch origin, living on average 25.5 years in the Netherlands (*sd* 11.4). About 25 % had a household income <€1000/month, and 26.6 % had a household income <€1350/month. Nearly half had low educational levels (48.6 %). The majority were not professionally employed (88.1 %).

Mean score on the health-related visual analogue scale (EQ-VAS scale 0–100) was 70.2 (*sd* 15.7), indicating reasonably good perceived health. Mean score for life satisfaction (scale 0–10) was 7.8 (*sd* 1.5). Most participants had a weak (34.3 %) or moderate (51.4 %) SoC, and 14.3 % had a strong SoC. Mean score on the scale for self-efficacy (scale 6–30) was 22.6 (*sd* 5.9), indicating fairly high levels of self-efficacy. Mean score on the Physical Activity Enjoyment Scale (scale 9–45) was 14.0 (*sd* 6.0), indicating high levels of physical activity enjoyment. About half of the respondents (52.8 %) participated <3 months in the CBHEPA programs, 47.2 % participated more than 3 months. The majority (68.9 %) exercised once a week, 28.5 % exercised more frequently. Fifty percent of the respondents paid a membership fee for the CBHEPA program, 50 % participated for free (Table 2). Membership fees ranged from €2.50 to €15.40, with an average of €6.95 (*sd* €4.64).

**Table 2:** Characteristics of WTP respondents

Variable	Value
*Predictors relating to personal conditions*	
Gender (n = 268)	
Women	86.6 %
Men	13.4 %
Age (n = 253)	
Mean (sd)	58.6 (14.0)
Range	26.64–90.64
Ethnic origin (n = 268)	
Dutch	35.4 %
Non-Dutch^a^	64.6 %
*Predictors relating to socio-economic conditions*	
Income (n = 256)	
< €1000	25.4 %
€1001–€1350	26.6 %
€1351–€1800	12.1 %
> €1800	7.8 %
Income not specified	28.1 %
Education (n = 256)	
No/primary education	48.6 %
Secondary education	42.4 %
College/university education	9.0 %
*Predictors relating to health-related quality of life conditions*	
EQ-VAS (0–100) (n = 259)	
Mean (sd)	70.24 (15.74)
Range	0–100
Life satisfaction (0–10) (n = 262)	
Mean (sd)	7.78 (1.49)
Range	1–10
Sense of coherence (SoC3) (n = 245)	
Strong SoC (3)	14.3 %
Moderate SoC (4–5)	51.4 %
Weak SoC (6–9)	34.3 %
Self-efficacy scale (n = 242)	
Mean (sd)	22.56 (5.85)
Range	8–30
*Predictors relating to sport and physical activity*	
Participation duration in CBHEPA program (n = 254)	
<3 months	52.8 %
3–6 months	15.4 %
>6 months	31.9 %
Frequency (n = 267)	
<1× week	2.6 %
1× week	68.9 %
2× week	19.1 %
>2× week	9.4 %
Physical Activity Enjoyment Scale (n = 250)	
Mean (sd)	14.04 (5.98)
Range	9–44
(Former) sports club member (n = 245)	
Yes	59.2 %
No	40.8 %
Leisure-time physical activity yes/no/(n = 265)	
Yes	85.3 %
No	14.7 %
Leisure-time sport yes/no (n = 264)	
Yes	42.8 %
No	57.2 %
Membership fee yes/no (n = 267)	
Yes	50.2 %
No	49.8 %

^a^Number of countries of origin: 29

### Willingness to pay for sport and physical activity

The average monthly WTP_money_ was €9.6 (*sd* 10.6) (Table 3). Variation in responses was fairly large. Over 16 % of the respondents were not willing to pay at all for sport and physical activity, mostly respondents in free CBHEPA programs. A little over 25 % were willing to pay to a maximum of €5/month, 45.5 % between €6 and €20; 13.0 % were willing to pay more than €20. The maximum WTP_money_ reported was €80 (n = 1). The average WTP_time_ was 17.6 min (*sd* 15.1) single journey travel time (Table 3). Two-thirds reported a maximum willingness to travel of between 5 and 20 min. The maximum WTP_time_ reported was 120 min (n = 1) to attend competition matches.

**Table 3:** WTP for sport and physical activity across groups

Variable	Amount	Respondents (%)
WTP_money_ (€/month) (n = 261)	€0	16.4
	€0–1	3.1
	€2–5	22.1
	€6–10	19.5
	€11–15	16.8
	€16–20	9.2
	>€20	13.0
	Mean (sd)	9.6 (10.6)
	Median	7.5
WTP_time_ (min/single-journey) (n = 246)	0–1	2.0
	2–5	6.4
	6–10	16.9
	11–15	27.7
	16–20	17.3
	21–25	7.2
	>25	22.5
	Mean (sd)	17.6 (15.1)
	Median	12.5

### Factors predicting willingness to pay for sport and physical activity

The dependent ordinal variables WTP_money_ and WTP_time_ were entered in an ordered probit model in SPSS22. Predictors measured as ordinal or categorical variables were entered as factors, predictors measured as scale variables were entered as covariates. Cases with missing values were excluded from analysis.

As expected for WTP_money_ (n = 176), our findings showed that low income (<€1000) was negatively related to WTP_money_, whereas perceived health (EQ-VAS) was positively related to WTP_money_. We also found that duration (>3 months) and frequency of participation (1× week or more), actual or former leisure-time sport participation, and physical activity enjoyment were positively related to WTP_money_ (Table 4).

**Table 4:** Ordered probit estimates of predictors for WTP for sport and physical activity

Variable	WTP_money_	(n = 176)	WTP_time_	(n = 172)
	Estimate	sd	Estimate	sd
*Personal and socio-economic*				
Income				
<€1000	−0.750	0.434*	1.154	0.424***
€1001–€1350	−0.027	0.413	0.374	0.404
€1351–€1800	0.302	0.499	0.100	0.496
>€1800	0.381	0.665	0.197	0.656
Not specified	Reference group		Reference group	
Educational level (low)	0.040	0.315	−0.442	0.314
Age				
<50 years	−0.805	0.550	−0.935	0.549*
50–64 years	−0.508	0.521	0.064	0.518
65–75 years	−0.0131	0.543	0.317	0.547
>75 years	Reference group		Reference group	
Ethnic origin (Dutch or non-Dutch)	−0.621	0.426	0.401	0.413
*Health-related quality of life*				
EQ-VAS	0.016	0.010*	0.013	0.010
Life satisfaction	0.004	0.099	0.128	0.096
Sense of coherence (SoC3)				
Weak SoC	0.325	0.511	−0.222	0.500
Moderate SoC	0.250	0.478	−0.302	0.457
Strong SoC	Reference group		Reference group	
Self-efficacy scale	−0.032	0.030	0.006	0.031
*Sport and physical activity*				
Duration of participation				
<3 months	−0.849	0.435*	−0.181	0.391
3–6 months	−0.684	0.516	0.046	0.496
6–12 months	0.337	0.539	−0.851	0.560
>1 year	Reference group		Reference group	
Physical Activity Enjoyment Scale	−0.048	0.026*	0.035	0.026
Frequency				
<1× week	−2.920	1.152**	−0.199	0.892
1× week	−0.297	0.518	−0.650	0.525
2× week	−0.351	0.546	−0.458	0.538
>2× week	Reference group		Reference group	
Leisure-time physical activity (no)	−0.098	0.478	−0.713	0.475
Leisure-time sport (no)	−0.604	0.315*	−0.419	0.317
Sports club membership				
(Former) member	−0.801	0.344**	−0.361	0.339
Never	Reference group		Reference group	
Membership fee (no)	−0.064	0.362	–	–
−2Log Likelihood	548.914		558.589	
Nagelkerke’s pseudo R^2^	0.393		0.199	

* *p* < 0.10; ** *p* < 0.05; *** *p* < 0.01

Contrary to our expectations, we found no relationships between educational level or ethnic origin and WTP_money_, between life satisfaction, self-efficacy or SoC and WTP_money_, and no relationship between leisure-time physical activity and WTP_money_ (Table 4).

As expected for WTP_time_ (n = 172), our findings showed that low income (<€1000) was negatively related to WTP_time_. Contrary to our expectations, age was positively related to WTP_time_. People younger than 50 years of age were less willing to travel for a longer time than people over 50 years of age. Contrary to our expectations, other personal and socio-economic predictors, the health-related and the sport and physical activity-related predictors did not seem relevant for predicting WTP_time_ (Table 4).

In sum, sport and physical activity program-related predictors were more relevant for predicting WTP_money_ than socio-economic or health predictors. Also, leisure-time physical activity did not seem relevant. For WTP_time_, only two of the socio-economic predictors, income and age, seemed to be relevant. Young age is related to lower WTP_time_. The expectation is confirmed for the lowest income level (<€1000) that income predicts WTP in terms of time and money. Educational level and ethnic origin seem unrelated to WTP, as well as sense of coherence, leisure-time physical activity, and paying membership fee (Table 5).

**Table 5:** Summary of results for WTP for sport and physical activity

Cluster	Predicting factor	Expectation	Who will be more likely to spend money on sport and physical activity?	Who will be more likely to spend travel time on sport and physical activity?	Expectation WTP accepted
Personal and socio-economic	Income	1. Income is positively related to WTP	Those with household incomes higher than €1000/month	Those with household incomes higher than €1000/month	Yes WTP_money/time_
	Educational level	2. Educational level is positively related to WTP	No difference between no, low, or high educational levels	No difference between no, low, or high educational levels	No
	Age	3. Age is negatively related to WTP	No difference between younger and older age	Those who are over 50 years of age	No, significant in opposite direction WTP_time_
	Ethnic origin	4. Non-Dutch origin is negatively related to WTP	No difference between those of Dutch and non-Dutch origin	No difference between those of Dutch and non-Dutch origin	No
Health and wellbeing	Perceived health status	5. Individual perceived health status is positively related to WTP	Those who score higher on perceived health status	No difference between those who score low or high on perceived health status	Yes WTP_money_
	Life satisfaction	6. Life satisfaction is positively related to WTP	No difference between those who score low or high on life satisfaction	No difference between those who score low or high on life satisfaction	No
	Sense of coherence	7. Sense of Coherence is positively related to WTP	No difference between those who score low or high on SoC	No difference between those who score low or high on SoC	No
	Self-efficacy	8. Self-efficacy is positively related to WTP	No difference between those who score low or high on self-efficacy	No difference between those who score low or high on self-efficacy	No
Sport and physical activity program	Duration	9. Duration of participation in the CBHEPA program is positively related to WTP	Those who participate more than three months	No difference between those who participate a shorter or longer period	Yes WTP_money_
	Frequency	10. Frequency of participation is positively related to WTP	Those who participate once a week or more	No difference between those who participated less or more frequently	Yes WTP_money_
	Physical activity enjoyment	11. Physical activity enjoyment is positively related to WTP	Those who score higher on physical activity enjoyment	No difference between those who score low or high on physical activity enjoyment	Yes WTP_money_
	Leisure-time physical activity	12. Additional leisure-time physical activity is positively related to WTP	No difference between those who are or are not additionally physically active in leisure time	No difference between those who are or are not additionally physically active in leisure time	No
	Leisure-time sport	13. Additional sport in leisure-time is positively related to WTP	Those who do additional sport in leisure time	No difference between those who do or do not do additional sport in leisure time	Yes WTP_money_
	Sports club membership	14. (Former) sport membership is positively related to WTP	Those who are or used to be engaged in sport	No difference between those who are or did not used to be engaged in sport	Yes WTP_money_
	Membership fee	15. Paying membership fee is positively related to WTP	No difference between those who are or are not paying a membership fee for the CBHEPA program	No expectation tested	No

## Discussion

We conducted this study to assess the WTP for sport and physical activity of participants in CBHEPA programs targeting socially vulnerable groups, expressed in money and time. Furthermore, we explored which factors predict WTP for sport and physical activity. We found relatively low WTP_money_ values, with a monthly average of <€10. This can be explained by the fact that around half of our study population represent, as intended, the lowest income levels in the Netherlands (Statistics Netherlands 2014). WTP research indicates that WTP is associated with a person’s ability to pay, in other words, person’s income (Donaldson 1999; Remonnay et al. 2008; Romé et al. 2010). The fact that particularly the lowest income category (<€1000) relates negatively to WTP suggests that the association between WTP for sports and physical activity in higher income groups might be more strongly related to other factors.

Respondents’ average WTP_time_ is around 17 min of single journey travel time. Our findings are consistent with other studies. A Dutch study reported a value for willingness to travel to sport facilities of 15 min (Prins et al. 2010). A German study reported values for willingness to travel ranging from 16 to 35 min among adult sports consumers (Pawlowski et al. 2009). This same study suggests that willingness to travel is related to type of sport and competition enrolment, and to how people prioritise their sport and physical activities.

In selecting variables to include in this study, we expected that predictors of health-related quality of life and physical activity behaviour would also predict WTP for sport and physical activity. However, we found several differences. As expected, the *personal and socio-economic predictors*, income and age, are related to WTP_money_. Low income (<€1000) is significantly negatively related to both WTP_money_ and WTP_time_. However, contrary to our expectations and findings of other studies (Krupnick et al. 2002), age (<50 years) is negatively related to WTP_time_. Probably, younger people face higher opportunity costs, i.e. benefits that could have been gained from an alternative use of the same resources (time and money) (Pampel et al. 2010), having to balance their time between household obligations, work, and leisure time. We did not find a relationship with other personal and socio-economic predictors, educational level or ethnic origin.

Of the* health-related quality of life predictors*, we found that perceived health is positively related to WTP_money_. This is consistent with other studies (Donaldson and Shackley 2003; Borghi and Jan 2008). We did not, however, find a relationship between WTP and life satisfaction, self-efficacy, and coping abilities (SoC). As mentioned before, we included these factors because they are well-known predictors of health-related quality of life and physical activity behaviour (Bauman et al. 2002; Hagger et al. 2002). Possibly, the reciprocal relationships between these factors have clouded our analysis used to study their relation to WTP for sport and physical activity.

*Sport and physical activity*-*related predictors* are most strongly related to WTP_money_—in particular how long and how often people participate in the program—and leisure-time sport experiences. On the basis of social cognitive theory, it can be argued that people who are or were members of a sports club haveknowledge of and experiences with sport. They might have more positive attributions to sport (Humpel et al. 2002; Nickel and Spink 2010) and are used to paying for sport (Higgins and Scholer 2009).

Our findings also indicate that respondents’ WTP_money_ exceeds the actual membership fee by approximately one-third (€2.64). This suggests that socially vulnerable groups attribute positive value to sport and physical activity in CBHEPA programs (Morris et al. 2007). On the other hand, we found a substantial percentage (16 %) of participants not willing to pay at all for sport and physical activity, in particular those enrolled in free CBHEPA programs. Future research could explore further whether or not respondents’ characteristics differ between those who were willing to pay and those who were not.

It may be argued that short-term program satisfaction is probably more decisive for WTP_money_ than long-term perspectives of improved health. Our findings indicate a possible time preference effect, i.e. an individual’s preference balancing between direct satisfaction from certain behaviour versus possible negative health consequences in the future (Jusot and Khlat 2013). Socially vulnerable groups generally show higher time preferences, focusing substantially on their wellbeing in the present, than high SES groups who place more emphasis on their wellbeing in the future (Chapman 2005). In this respect, our findings suggest that sport and physical activity program-related predictors best explain WTP for sport and physical activity, since these relate to actual physical activity experiences and short-term benefits. Physical activity enjoyment is an example of such a short-term benefit, as opposed to other positive health benefits (i.e. weight loss), which are future gains and therefore hard to predict (Dacey et al. 2003; Henderson 2009; Mullen et al. 2011). Our findings are consistent with research by Romé et al. (2010), who concluded that people report the highest WTP for immediate health improvements.

Assessment of WTP is presented in the health economics literature as a relatively easy method to study perceived benefits at individual level of health-related quality of life interventions in different communities and different contextual settings (Bayoumi 2004). Compared to assessing quality-adjusted life years (QALYs), estimating individual WTP has indeed some advantages, as stated in the literature: (1) WTP is theoretically grounded in welfare economics, (2) WTP does not need specification of which parts of the intervention need to be valued by respondents, and (3) WTP values express benefits in monetary terms (Donaldson et al. 1997; Olsen and Smith 2001; Shackley and Donaldson 2002). We faced, however, some methodological challenges in assessing WTP in socially vulnerable groups. First, about 16 % of our respondents are not willing to pay for sport and physical activity, and the lowest income level is negatively related to WTP, indicating that answers are probably more reflective of people’s actual income positions than of their willingness to pay (Hagberg and Lindholm 2006). As a result, our study might underestimate rather than overestimate WTP_money_ values. Second, Hagberg and Lindholm (2006) state that less educated respondents may show less understanding of the real and hypothetical situations as examined in WTP. This is consistent with our observations during the study, in which respondents occasionally seemed unable to distinguish between what they could afford and what they were willing to pay for sport and physical activity. It is also consistent with the negative relationship we found between WTP and low income. Third, respondents may have responded strategically in the hope that their answers would influence the actual pricing of their CBHEPA programs, as has been found in other studies (Smith 2003; Morris et al. 2007).

We addressed the methodological challenges by using closed-ended WTP questions. As the WTP data collection was integrated in a more comprehensive questionnaire to evaluate CBHEPA program outcomes, we tried to keep questions concerning different topics as concise and clear as possible, in view of our target group. Questionnaire use can be difficult in socially vulnerable groups. Lack of health literacy, lack of basic skills in reading and writing, and different beliefs about (health) concepts across cultures may lead to difficulties in understanding and interpreting the questions (Bonevski et al. 2014), eventually leading to non-response (Feskens et al. 2006). Our approach contributed to clarity and uniformity of data collection procedures within and between groups. In line with recommended procedures for WTP data collection, suggested by Smith (2003), offering the necessary specifications of the context and the service that people are valuing, our data collection in context, i.e. during the exercise class, contributes to the methodological robustness of our WTP study. On the other hand, our predefined WTP response categories may have limited people’s choice. Group-wise data collection may also have had an impact on individual WTP responses.

## Conclusion

Our assumptions that factors predicting health-related quality of life and WTP for health improvements may be relevant for predicting WTP for sport and physical activity are not unequivocally supported in this study. People from socially vulnerable groups, active in CBHEPA programs, are willing to pay for sport and physical activity, albeit low amounts. WTP in terms of money is significantly related to income and (former) experiences in sport and physical activity. WTP in terms of travel time is significantly related to income and age. Our findings for WTP for sport and physical activity are in line with studies reporting that WTP is not responsive to changes in health over time, indicating that health improvements over time do not simply result in a positive change in WTP (Harris et al. 2013). Income and short-term program satisfaction are probably more decisive for WTP_money_ than long-term perspectives of improving health-related quality of life. Awareness of these factors predicting WTP could contribute to future policy and development of CBHEPA programs, focusing on service provision to enhance people’s behavioural competences for physical activity maintenance and program satisfaction rather than aiming at long-term health improvements.

## Abbreviations

CBHEPA: community-based health enhancing physical activity
CoM: communities on the move
CVM: contingent valuation method
EQ-VAS: EuroQoL visual analogue scale
NISB: Netherlands Institute for Sport and Physical Activity
QALY: quality-adjusted life years
SES: socio-economic status
SoC: sense of coherence
SQUASH: Short Questionnaire for Sport and Physical Activity
WTP: willingness to pay
